# A data driven approach to mineral chemistry unveils magmatic processes associated with long-lasting, low-intensity volcanic activity

**DOI:** 10.1038/s41598-023-28370-0

**Published:** 2023-01-24

**Authors:** Simone Costa, Luca Caricchi, Marco Pistolesi, Anna Gioncada, Matteo Masotta, Costanza Bonadonna, Mauro Rosi

**Affiliations:** 1 Dipartimento Di Scienze Della Terra, Università Di Pisa, Via S. Maria, 53, 56126 Pisa, Italy; 2 Department of Earth Sciences, University of Geneva, Rue Des Maraîchers 13, 1205 Geneva, Switzerland

**Keywords:** Petrology, Volcanology, Petrology, Volcanology

## Abstract

The most frequent volcanic eruptions are of low-intensity and small magnitude. They produce abundant ash-sized (< 2 mm) clasts, which are too small to establish quantitative links between magmatic processes and eruptive dynamics using classic approaches. This inhibits our ability to study the past behaviour of frequently erupting volcanoes, essential to predict their future activity and mitigate their impact. The Palizzi unit (10–13th century, Vulcano, Italy) includes a prototype sequence of ash deposits resulting from prolonged Vulcanian eruptions punctuated by those of two larger sub-Plinian events. We apply Hierarchical Clustering to chemical analyses of clinopyroxene collected along the stratigraphy to decipher magma dynamics during this eruptive period. We identify periods of magma input and we link deep magmatic processes to eruptive dynamics, also showing that our approach can be used to connect magma and eruptive dynamics in any volcanic sequence. This is essential to track the processes occurring during frequent eruptions and to identify the build-up to larger explosive events.

## Introduction

Fine-grained fallout tephra beds, resulting from low-intensity, magmatic explosive eruptions, are complex to characterize by petrological and textural analysis with respect to lapilli-sized material or lava samples. In addition to the difficulty of separate juvenile glass and crystals from non-juvenile components, the bulk magma composition is complicated to obtain due to the selective atmospheric transport of glass and crystals of different density^1,2^. For this reasons, bulk-rock chemistry of the ash deposits can vary with distance from the vent and more in general it does not necessarily represent the juvenile magma that fed the eruption^1,2^. The deposits of long-lasting, low-intensity eruptions (VEI 1–2) are the most frequent (1–10 yrs) and/or the most easily accessible in the eruptive record^3–6^. Therefore, the difficulty we encounter in characterising these deposits jeopardises our capacity to trace the relationships between magmatic processes and eruptive dynamics over the eruptive history of any volcanic system. In addition, long-lasting, low-intensity eruptions cause a continuous threat to communities living close to active volcanoes (e.g. Sakurajima volcano, Japan^7^) and following their temporal evolution is essential to determine whether the style of activity might change in future. However, their investigation with classic approaches would require the collection of samples in proximal areas (coarse material), which either relies on special equipment such as Remote-Control Vehicles or could be otherwise extremely risky (e.g. 2021 eruption of Cumbre Vieja eruption, La Palma, Spain^8^). Some ash deposits do not even have their coarse counterpart in proximal area, in which case, petrological analysis would be just impossible.

**Figure 1:**
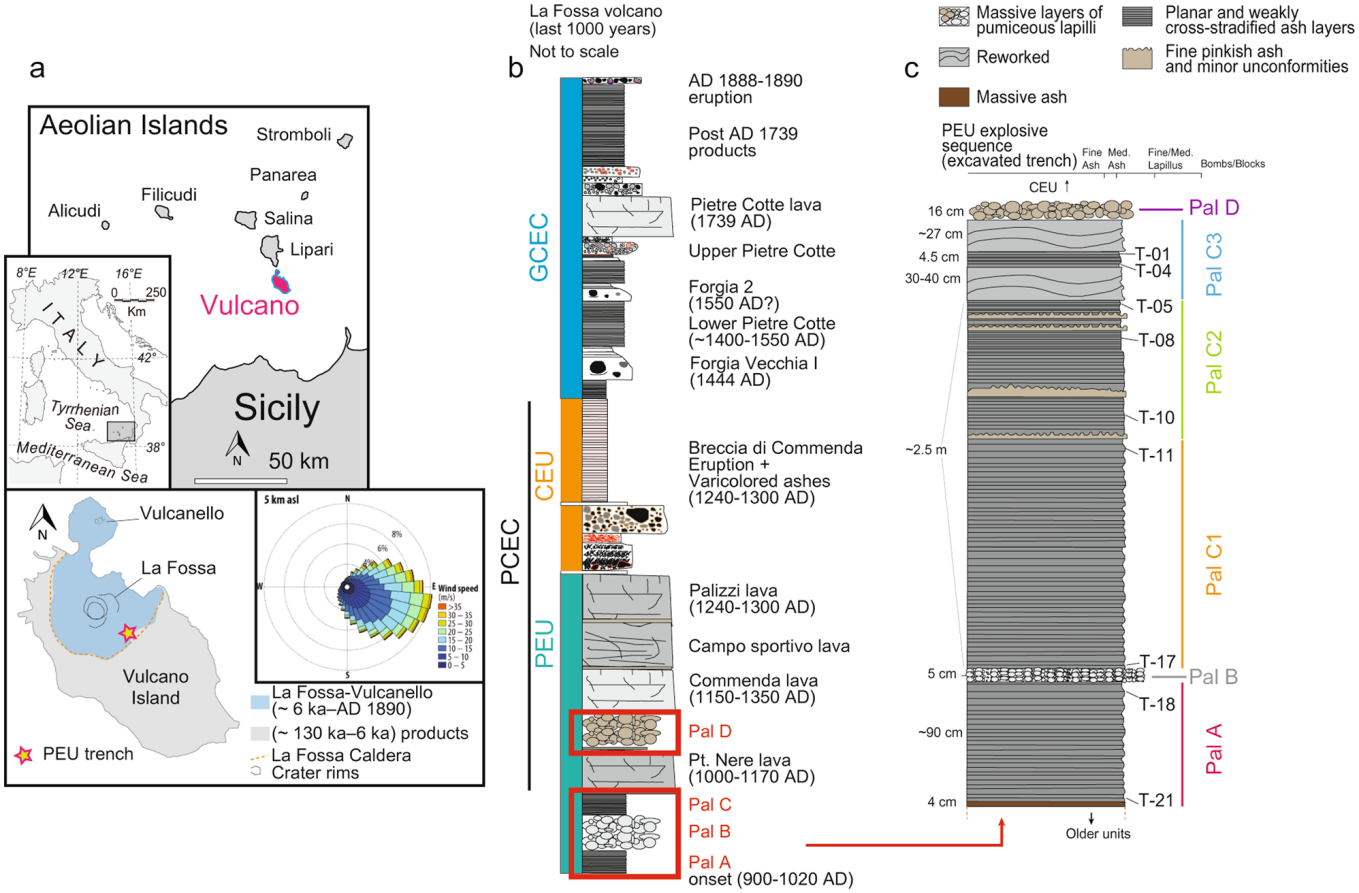
Location of Vulcano Island and La Fossa stratigraphy of the last 1000 years. (**a**) Location of Vulcano Island in the Aeolian Arc (Southern Italy) and simplified geological sketch map showing the position of the excavated trench where the PEU has been sampled (Methods); rose diagram in the insert showing the prevalent wind conditions at 5 km above sea level is from ref.^28^ .(**b**) Eruptive stratigraphy of the last 1000 years (after ref.^26,27^) is summarized in two main eruptive clusters: the Palizzi-Commenda eruptive cluster (PCEC), developed during the 10-fourteenth centuries, and the Gran Cratere eruptive cluster (GCEC, fifteenth century-1890 AD). The stratigraphic sequence of PCEC encompasses the products belonging to the Palizzi eruptive unit (PEU) and of the Commenda eruptive unit (CEU). The PEU consists of (i) cross-stratified and parallel-bedded ash layers (Pal A and Pal C); (ii) pumiceous lapilli fallout layers of Pal B rhyolite and Pal D trachyte; (iii) lava flows, including the obsidian Commenda lava and the trachytic lava flows of Palizzi, Campo Sportivo and Punte Nere; chronological constraints are from ref.^27^ and references therein. (**c**) Simplified stratigraphic log of the explosive sequence of the PEU showing the layers sampled in this work. (see also Supplementary Information and Fig. S1 for additional details about stratigraphic features). Maps in (**a**) were generated using the Generic Mapping Tools software (Release GMT 6.0.0; www.generic-mapping-tools.org) by Simone Costa; maps in (**a**) were modified using Adobe Illustrator (Release 2022, 26.3.1; www.adobe.com), and drawings in (**b**) and (**c**) were prepared using the same software by Simone Costa.

The analysis of zoning patterns of crystals, both in explosive and effusive products, provides insights into the time scales and processes that occur within the plumbing system, such as the evolution and variation of the melt chemistry and changes of the intensive parameters during crystal growth^9,10^. In recent years, the development of machine learning algorithms has greatly improved the analysis of a large number of petrologic data expanding our ability to identify links between magmatic processes, eruptive style and monitoring signals^11–25^. In this work, we develop a workflow based on unsupervised machine learning (Hierarchical Clustering; HC) that allows to investigate the magma dynamics associated with explosive sequences dominated by fine-grained material. We focus on the explosive products of the Palizzi Eruptive Unit (PEU) of La Fossa volcano^26^ (Vulcano Island, Italy; Figs. 1a–c), as an emblematic example of this kind of sequences. The PEU (10–13th century)^27^ represents one of the most important eruptive periods of La Fossa volcano and is characterized by variable eruptive dynamics that produced a large spectrum of tephra-fallout deposits^26^. Given the fine grain-size of the available outcrops, the PEU pristine and complete sequence has remained poorly explored from a petrologic point of view. The tephra sequence has the undeniable value of providing an orderly temporal complete succession of eruptive materials, being located in the downwind sector with respect to the vent^28^ and on a topographic relative high which protects it from erosive factors (Figs. 1a and S1; Methods and Supplementary Information).

In this study, we use major element analyses collected using an electron probe microanalyzer (EPMA) along core-to-rim transects in clinopyroxene (cpx) crystals to perform HC and reduce the cpx profiles to a sequence of clusters^17^ and objectively quantify chemical and textural variations along the PEU sequence. Cpx is stable over a wide range of P–T–H_2_O and thus is prone to capture the variation of physical and chemical conditions within the volcanic plumbing system over time^29–37^. We then couple HC with cpx-melt thermo-barometry and use clusters distribution to identify petrological proxies for deciphering the complex relationship between cpx chemical zoning and magma recharge events, possibly related to the build-up phase to larger explosive eruptions. Using this approach, we show that it is possible to associate the eruptive dynamics, inferred along the stratigraphy of ash-dominated volcanic deposits, to magmatic processes occurring at depths.

## Results and discussion

### The PEU explosive products

The fine-grained PEU tephra sequence has been interpreted as the result of persistent Vulcanian explosive activity and was accumulated at about 1 km from the vent^26^. Tephra deposits mostly consist of ash layers (Pal A and Pal C^26^, further divided in this work in the sub-units Pal C1, Pal C2 and Pal C3) and punctuated by two lapilli layers related to sub-Plinian eruptions (Pal B and Pal D)^26–28,38–40^ (Figs. 1b, c and S1; Methods and Supplementary Information). Ash layers of Pal A and Pal C1 are plane-parallel suggesting they were accumulated in rapid succession by namely fallout activity; Pal C2 and Pal C3 contain an increasing number of intra-sequence erosive unconformities, suggesting Vulcanian explosions with longer repose intervals, as also highlighted by the occurrence of two levels of reworked deposits in Pal C3. Pal B and Pal D are lapilli- to bomb-sized layers with constant, pluri-cm thickness from fallout activity (Figs. 1c and S1).

**Figure 2:**
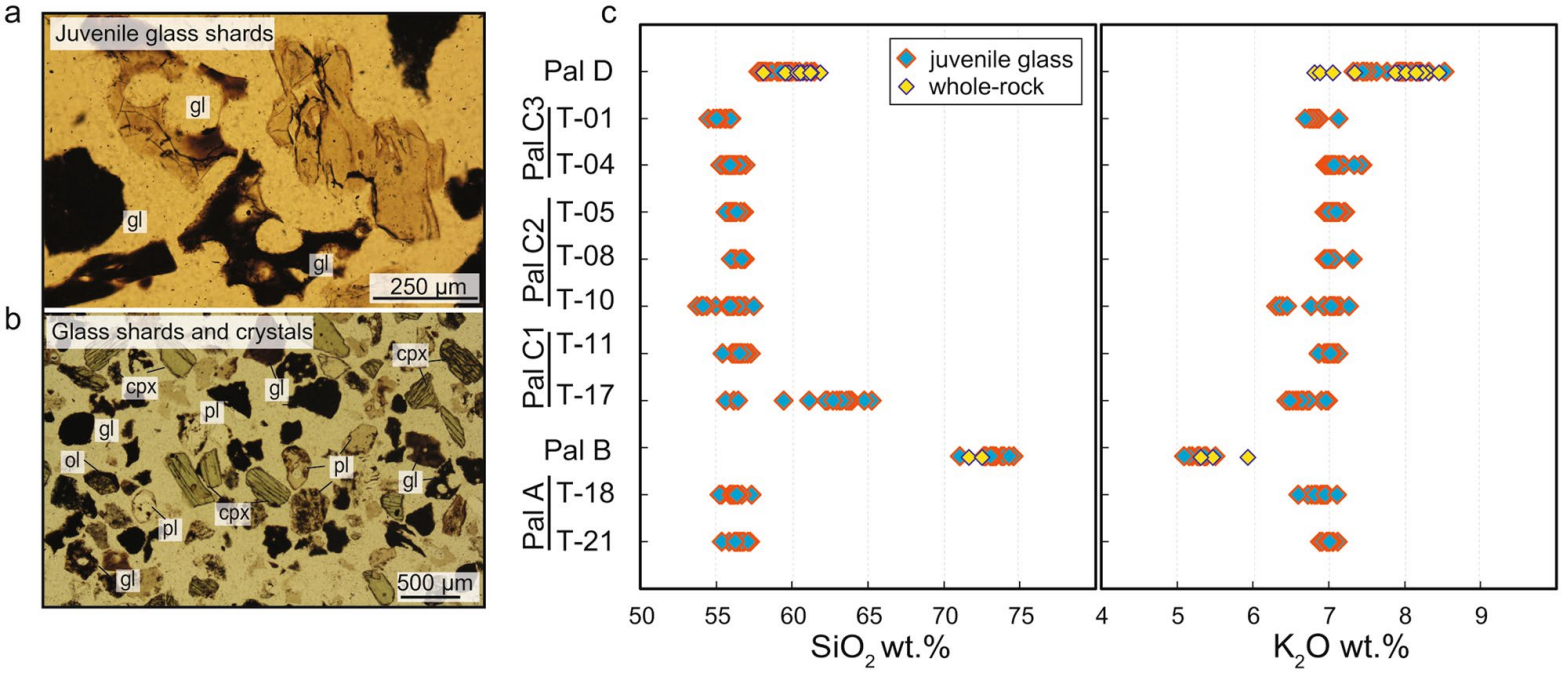
Petrographic features and major element chemical composition of the PEU explosive products**.** (**a**)Microphotograph showing vesiculated transparent to opacified juvenile glass shards in the PEU ash layers. (**b**) Phenocrysts in the PEU ash layers; cpx: clinopyroxene, pl: plagioclase, ol: olivine, gl: glass shards. (**c**) Chemo-stratigraphy of SiO_2_ and K_2_O (wt.%) showing whole-rock data of Pal B and Pal D (data from ref.^38,44,46^), and juvenile glass fragment analyses of Pal A, Pal C and and matrix glasses of Pal B and Pal D (EPMA data from this work).

The petrographic inspection of the PEU ash layers (Pal A, Pal C1, Pal C2 and Pal C3) highlights the presence of five main categories of components: (i) juvenile vesiculated glass, light brown in colour, both transparent and opacified (Fig. 2a), (ii) fresh to altered lithic fragments and (iii) loose crystals, mostly euhedral to subhedral clinopyroxene, plagioclase and minor amount of olivine (Fig. 2b). The Pal B deposit consists of coarser-grained, white pumiceous lapilli and bombs bearing a limited amount of crystals (P.I. < 5 vol.%) set in a glassy groundmass^38^. Phenocrysts are, in order of abundance, plagioclase, sanidine, clinopyroxene and biotite. The Pal B pumice also hosts latitic enclaves constituted by phenocrysts of plagioclase and clinopyroxene in a dark microcrystalline matrix^38^. The Pal D fallout layer is constituted by highly vesiculated (~ 80 vol.%) and low porphyritic (P.I. < 5 vol.%) lapilli- to bomb-sized pumice clasts^38,40^. Millimetric phenocrysts of plagioclase, clinopyroxene, sanidine, biotite and olivine occur in a brown glassy groundmass^38,40^.

The overall small grainsize of the PEU ash layers did not allow bulk-rock analyses of the erupted tephra, and only the composition of the juvenile glass for the different levels was obtained. Instead, whole-rock data have been acquired for the largest intensity eruptions (Pal B and Pal D; Fig. 2c). Pal B pumice clasts are rhyolitic^38^ in composition (Fig. 2c) whereas those from Pal D are trachytic and show the highest K_2_O content of Vulcano products (K_2_O up to ~ 7.6 wt.%) and SiO_2_ contents comparable to other latitic and trachytic magmas emitted at La Fossa (~ 58 to 61 wt.% SiO_2_; Fig. 2c)^38,40^. The groundmass glasses of Pal B and Pal D show similar composition to the respective whole-rock analyses (Fig. 2c). Pal A, Pal C1, Pal C2 and Pal C3 show moderate variations in the juvenile glass composition from the base to the top of the sequence, with SiO_2_ in the range ~ 53.5 to 57.4 wt.% and 6–7.4 wt.% K_2_O. Only the juvenile glass of a specific Pal C1 sample (T-17), the first level after the emplacement of the Pal B rhyolite, covers a wider silica range spreading to silica-rich compositions (55.6–65.2 wt.% SiO_2_ and 6.4–6.9 K_2_O wt.%; Fig. 2c). Groundmass glasses compositions by EPMA are reported in the electronic Supplementary Material.

### Textural and chemical features of clinopyroxene

**Figure 3:**
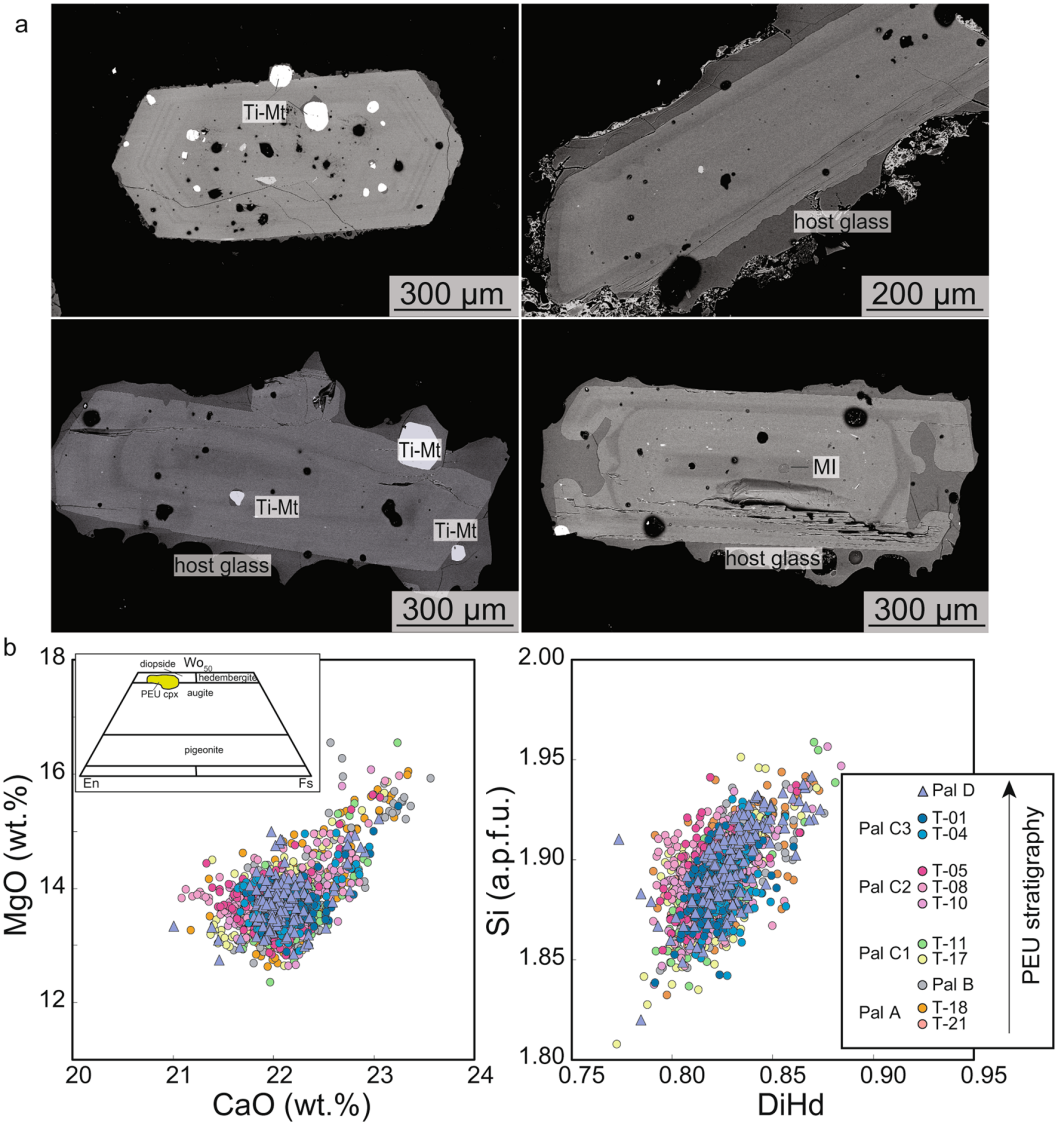
Textural and chemical features of clinopyroxene. (**a**) BSE images of clinopyroxene crystals of PEU, MI: melt inclusion, Ti-Mt: Ti-magnetite. (**b**) compositional diagrams of analyzed clinopyroxene; DiHd: Dipside-Hedembergite components; a.p.f.u.: atoms per formula unit.

Back-scattered electron (BSE) images of cpx crystals belonging to the PEU explosive products show different zoning patterns, ranging from un-zoned to weakly concentrically zoned (Fig. 3a). Oscillatory zoning is sometimes present, as testified by concentric bands with variable BSE intensity. In several cases, cores are characterized by lower BSE intensity, whereas sector zoning has not been observed. Inclusions of Ti-magnetite, apatite and glassy melt inclusions are commonly observed either in the cores or in concentrically zoned portions of the crystals (Fig. 3a). Although sector zoning is not revealed by BSE images, small compositional variations can occur between the basal {− 111} and prism {hk0} sectors. In order to minimize the effects of these intra-crystalline variations, cpx analyses were performed following core-to-rim profiles oriented from the center of the crystal, perpendicular to the c axis (note that all cpx crystals were exposed in section parallel to the c axis). Following this procedure, only prism sectors {hk0} are analysed, making the results of HC analysis and thermo-barometry self-consistent (see following sections and Methods). The composition of cpx from PEU covers a narrow range parallel to the augite-diopside joint (Wo_41-49_, En_35-47_, Fs_6-19_; Fig. 3b) and it is comparable with that of cpx occurring in shoshonitic and lati-trachytic magmas emitted in the last 1000 years at Vulcano^41,42^. The average chemistry of cpx is roughly constant along the entire stratigraphic sequence of PEU (Fig. 3b), which would suggest no significant variations of the pre-eruptive conditions over this eruptive period.

### Cluster analysis of clinopyroxene

**Figure 4:**
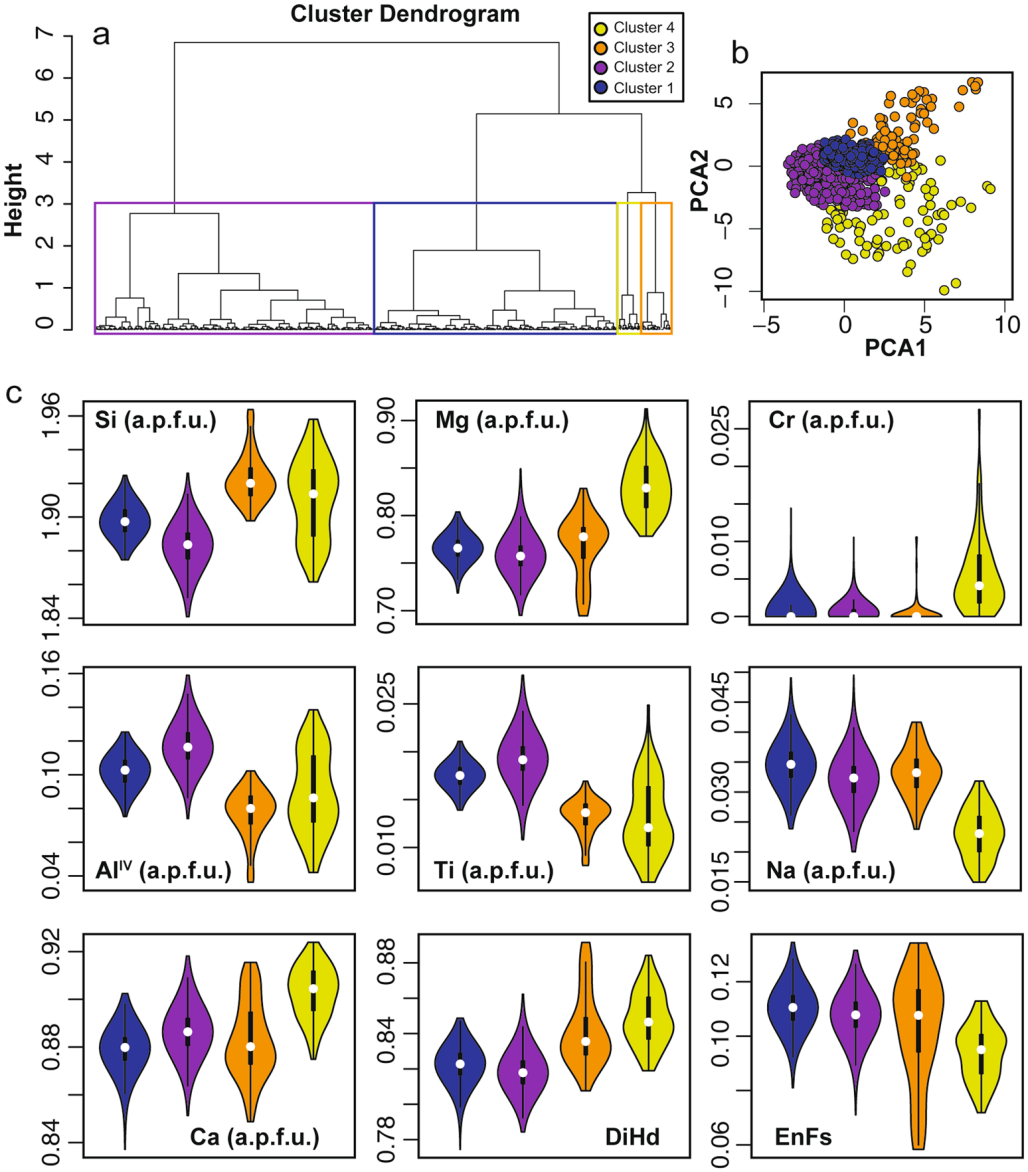
Cluster analysis results. (**a**) Dendrogram obtained by the Ward linkage clustering method (Methods). The coloured boxes show the groups of analyses associated with each cluster. The height represents the compositional difference required to merge two clusters together. (**b**) cpx clusters projected using the first (PCA1) and second (PCA2) principal components (Methods). (**c**) Violin plots showing the density distribution of the chemical composition of each cluster; a.p.f.u.: atom per formula units.

**Figure 5:**
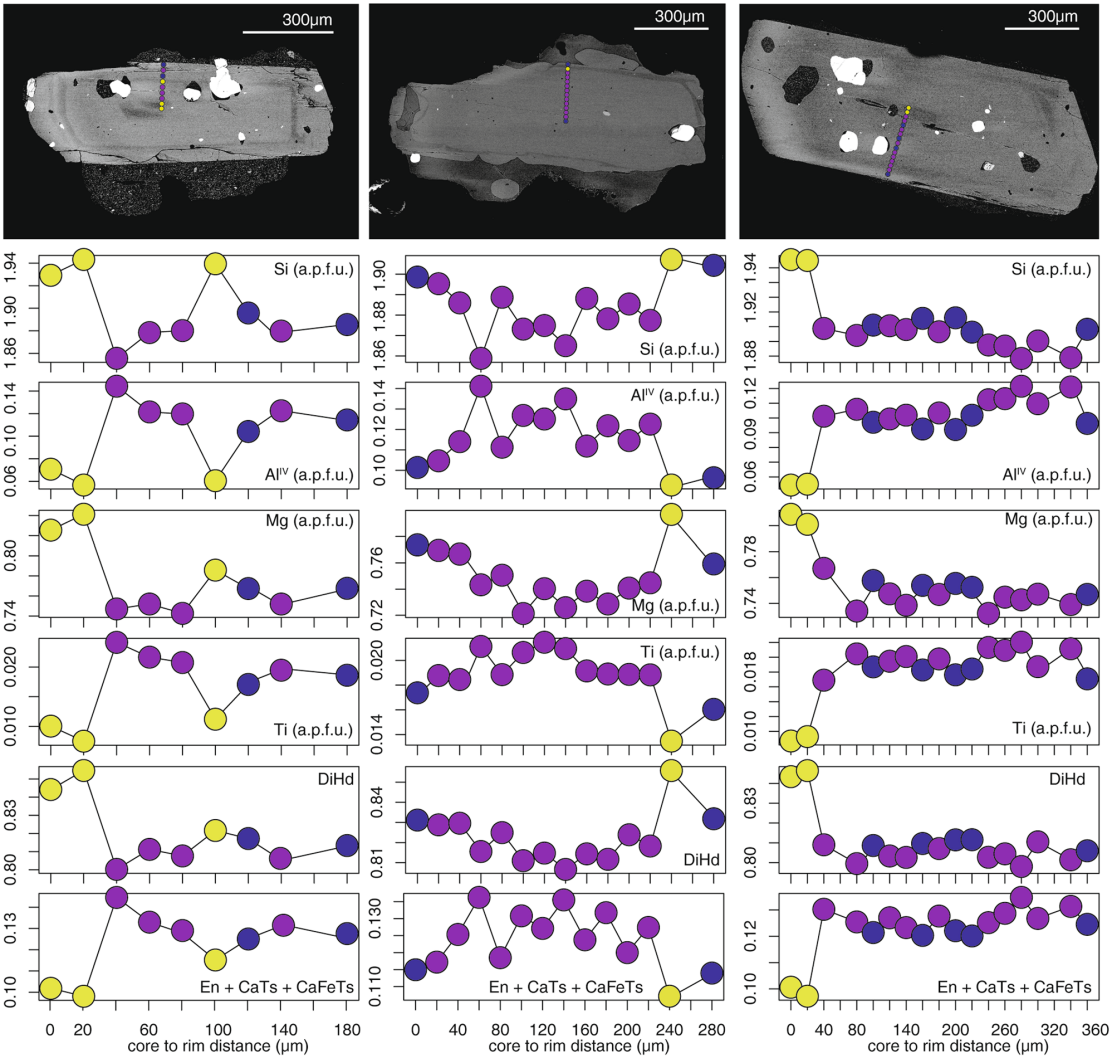
Clinopyroxene chemical profiles. Examples of clinopyroxene profiles with the textural position of clusters in BSE images; a.p.f.u.: atom per formula units.

**Figure 6:**
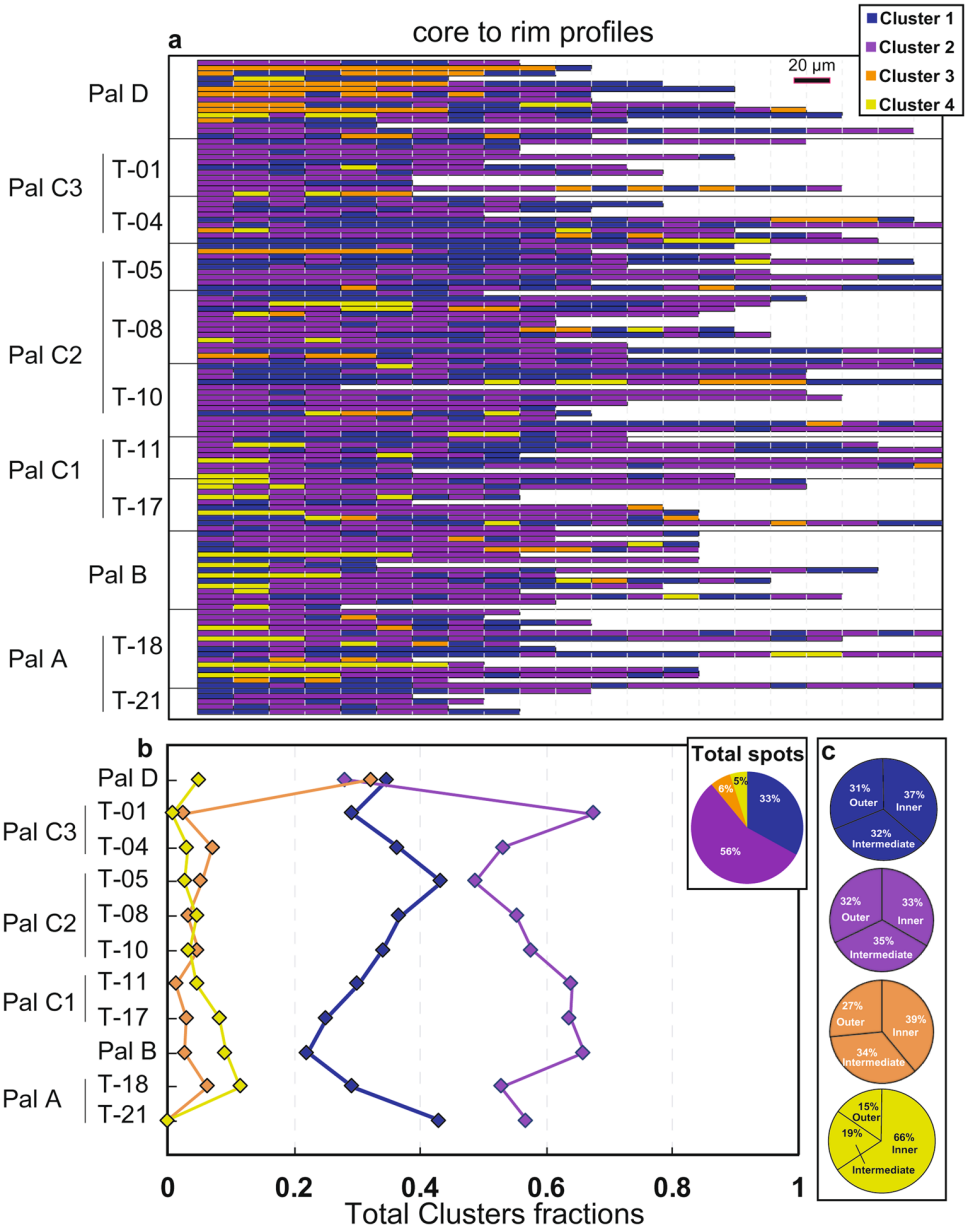
Distribution of cliopyroxene clusters along the stratigraphy of the PEU. (**a**) Barplot showing the total sequence of clusters for each collected chemical profile. Each segment represents an analytical spot spaced 20 µm. (**b**) Total distribution of clusters and cluster fractions for each stratigraphic level. (**c**) Total distribution of each cluster divided for inner zone (33%), intermediate zone (33%) and outer zone (33%), see text for additional explanation.

Hierarchical cluster analysis was conducted using log-transformed major elements chemical composition (e.g. ref.^25^; Methods) of cpx collected along the tephra sequence (Figs. 4a–c and S2), including the two main pumiceous deposits (data are reported in the electronic Supplementary Material). The clustering procedure allows to simplify the multidimensional complexity of cpx chemical profiles into a series of clusters^17^, with each data point assigned to a specific cluster (Figs. 5 and 6a). Additionally, this approach permits to easily quantify the relative proportions of clusters present throughout the investigated sequence (Figs. 6a, b and S3). The results show that the cpx of the PEU are best represented by four compositional clusters (Figs. 4a and S2; Methods). Plotting data with colours representing each cluster in the two principal components space show that the clusters identified by HC are well defined (Figs. 4b and S2; Methods).

Clusters 1 and 2 are similar for all elements, with the exception of Si and Mg that are slightly higher in cluster 1, and Al and Ti, slightly higher in cluster 2, respectively (Fig. 4c). Cluster 2 and subordinately Cluster 1 show the highest content of Ti and Al among all clusters. Cluster 3 shows an intermediate chemical composition between clusters 1 and 4 and has the highest Si content and the lowest concentration of Al, Ca and Cr. Cluster 4 shows the largest chemical differences with respect to the other three clusters being enriched in Si, Ca, Mg, Cr and having lower Fe and Na (Fig. 4c). It is worth noting that almost all the spots assigned to cluster 4 correspond to the low-intensity zones in BSE images, while it is more complex to identify relationships between the other clusters and textural features (Fig. 5).

Once identified the four clusters, it is possible to quantify their relative abundances; particularly, HC assigned the 56% of the total analytical spots to cluster 2, 33% to cluster 1, 6% to cluster 3 and 5% to cluster 4 (Fig. 6b). Cluster 1 is the most abundant at the base of the sequence (43%) and reaches its minimum in correspondence of Pal B (22%). Cluster 2 represents between 49 and 67% of the spots in all stratigraphic levels and reaches is minimum in correspondence to Pal D (28%; Fig. 6b). Cluster 3 is absent at the base of the sequence and always represents less than 7% of the analyses. The only exception is in Pal D where cluster 3 is more abundant and represents 32% of the spots. Cluster 4 is absent at the base of the sequence (base of Pal A) and reaches its maximum (9–11%) in the following levels (the top of Pal A and Pal B; Fig. 6b).

Each profile has been divided in three equal spatial portions (33% of the total length of the profile) to determine the distributions of clusters in different portions of the crystals, here defined as inner, intermediate and outer zones. In this respect, as we do not recalculate the relationships between the position of a specific zone and the relative volume of the zone, our approach serves to highlight relative temporal variation recorded by crystals that cannot be directly related to the volume of magma experiencing specific conditions. The relative abundance of the four clusters varies along the PEU stratigraphy as does their distribution between inner, intermediate and outer portions of cpx (Figs. 6c and S3). Cluster 1 and 2 are equally distributed in the three zones (Fig. 6c). Cluster 3 is slightly more abundant in inner (39%) and intermediate (34%) portions with respect to outer zones (27%) (Fig. 6c). Cluster 4 is most abundant in cpx inner portions (66%) with respect to intermediate (19%) and outer (15%) zones (Fig. 6c). Other interesting features are also observed when the total fractions of clusters are plotted along the stratigraphic sequence (Fig. 6b). To an increase of analyses relative to cluster 2 corresponds a decrease of the spots belonging to cluster 4. The distribution trend of cluster 1 is specular to that of cluster 2 (Fig. 6b). The same trends are observed when plotting the cluster fractions in the different crystal zones along the stratigraphy (Fig. S3).

### Thermo-barometry of clinopyroxene clusters

**Figure 7:**
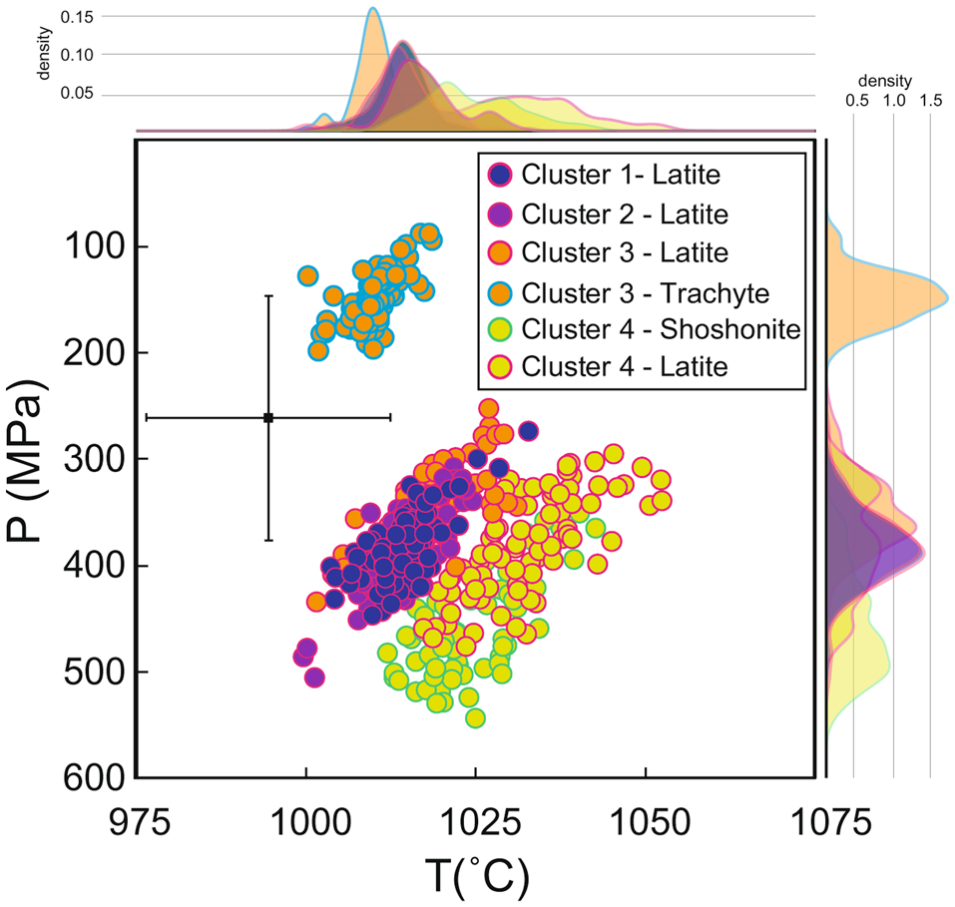
Thermo-barometry of clinopyroxene clusters. Pressure and temperature estimates obtained for the clinopyroxene clusters using the clinopyroxene-melt thermobarometer of ref.^36^ (the cross represents the P and T errors of the models). The density plots on the side of the diagram show the distribution of P and T calculations. See Methods section and Figs. S4 and S5 for cpx-melt equilibrium criteria.

Crystallization pressure and temperature have been determined using the cpx-melt thermo-barometer of ref.^36^ (Fig. 7), after checking the equilibrium condition between cpx and coexisting melt (Figs. S4 and S5 and Tab. S1; Methods). This thermo-barometric model is specific to alkaline-differentiated magmas and includes in the calibration dataset experiments performed with lati-trachytic products erupted at La Fossa volcano during the PEU^36^. All clusters have been paired with the average melt compositions of coeval shoshonitic and latitic magmas, while cluster 3 has been also paired with the trachytic composition of Pal D because it mainly occurs in cpx of this eruption (Figs. S4 and S5 and Tab. S1; see Methods for further explanation on cpx-melt equilibrium criteria). The H_2_O content required as input data for the thermo-barometer for the cpx-shoshonite pairs was varied between 0.3 and 0.6 wt.% (i.e. the H_2_O range in shoshonitic melt inclusions measured by ref.^42^). For cpx-latite pairs, H_2_O varied between 2.5 and 3.5 wt.% and between 1.5 and 2.5 wt.% for trachyte-cpx pairs (obtained through plagioclase-liquid hygrometry by refs.^38,43^ and H_2_O in melt inclusions by Raman spectroscopy^40^). We noticed that an increase in H_2_O content of 1 wt.% corresponds to a maximum decrease of temperature estimates of ~ 10 °C, which is lower than the uncertainty on temperature estimates (± 18 °C)^36^.

Crystallization temperature and pressure estimates for cpx crystals of clusters 1 and 2 latitic pairs are identical within the error, respectively 1014 ± 4 °C and 382 ± 31 MPa for cluster 1 and 1014 ± 5 °C and 386 ± 35 MPa for cluster 2. Cpx crystals of cluster 3 paired with a latitic composition show similar temperature and slightly lower pressure compared to the other clusters, respectively 1017 ± 5 °C and 351 ± 34 MPa; when paired with a trachytic composition, they show the lowest temperature and pressure ranges among all clusters, of 1010 ± 3 °C and 151 ± 23 MPa, respectively. Since cluster 3 mainly occurs in the trachytic products of Pal D and also given the equilibrium tests results (Figs. S4  and S5), we retain that cluster 3-trachytic melt pairs provide better estimates with respect to cluster 3-latite pairs. Finally, cpx crystals from cluster 4 paired with a latitic melt composition yielded temperature of 1033 ± 8 °C and pressure of 380 ± 48 MPa, whereas, coupled with a shoshonitic melt yielded temperature of 1024 ± 7 °C and pressure of 458 ± 48 MPa (Fig. 7).

In general, the inferred pressures for the cpx clusters-latite melt pairs agree with those inferred in previous works, based on cpx-melt thermobarometry and thermodynamic modelling^38,44–46^, for the shoshonitic to latitic reservoir (17–12 km) of La Fossa plumbing system, that has been active at least in the last 1000 years. Cpx crystals of cluster 4 coupled with shoshonitic melts (estimated pressure of 458 ± 48 MPa) suggest equilibration pressure within the shoshonitic reservoir inferred for the La Fossa-Vulcanello system at depths between 18 and 21 km through volatiles in melt and fluid inclusions in quartz xenoliths^44,47–49^. Finally, the pressure obtained for the cpx crystals of cluster 3 paired with a trachytic melt composition (151 ± 23 MPa), confirms the presence of a shallow storage region that fed the Pal D eruption as suggested through cpx-melt thermobarometry^38^. It should be noted that, with the exception of cluster 3-trachytic melt pairs, the range of estimated pressure and temperature falls within the error of the thermo-barometric models (18 °C and 115 MPa)^36^.

### Petrological interpretation of clinopyroxene clusters

Compositional zoning of cpx is typically associated with crystallization kinetics that, in turn, can be related to the varying degree of undercooling^50–57^. Diffusion-controlled growth mechanism prevails with increasing degree of undercooling, yielding to incorporation of chemical species that are incompatible in the crystal structure^31^. In the case of cpx, enrichment in incompatible elements such as Al^IV^ and Ti can be interpreted as the result of crystallization under regimes of high undercooling, resulting from cooling and degassing processes^56^. The chemical differences among the cpx clusters can be therefore associated with different regimes of undercooling experienced during the growth of cpx crystals and to magma dynamics within the plumbing system (Fig. S6).

In terms of chemical composition of the clusters, the majority of analyses belonging to cluster 2 shows higher concentration of Al^IV^ and Ti with respect to other clusters (Fig. 4c). Excluding cluster 3 (which we discuss separately below), the concentration of Al^IV^ and Ti overall decreases from cluster 2 to cluster 1, and from cluster 1 to cluster 4 (Fig. 4c). This latter cluster, on the counterpart, results enriched in Si, Mg and also Cr (Figs. 4c, 5, S6). Cluster 4, which mainly occurs in cpx inner zones (66%; Figs. 5, 6c), could be thus related to slower crystallization under lower regimes of undercooling. Importantly, the higher content in Cr observed in cpx crystals from this cluster could be interpreted as evidence of crystallization from a more mafic magma^58^. This agrees with thermo-barometric calculations showing that cluster 4 is associated with crystallization at higher temperature and pressure with respect to other clusters (Fig. 7). On the other hand, the chemistry and the small compositional differences of clusters 1 and 2 could be interpreted as the consequence of a more rapid growth under higher and variable regimes of undercooling, possibly resulting from decompression-induced degassing^59^ and reflecting different moments of near-equilibrium crystal growth.

Concerning cluster 3, we note that cpx belonging to this cluster are mostly concentrated in the Pal D eruption and, consistently, they show a compositional variability that is not apparently correlated to the variations described for the other clusters. Accordingly, thermo-barometric calculations for cluster 3-trachyte melt pairs suggest crystallization at lower temperature and pressure conditions (Fig. 7) that are typical of the trachytic magma of Pal D^38^.

### Plumbing system processes during the PEU

**Figure 8:**
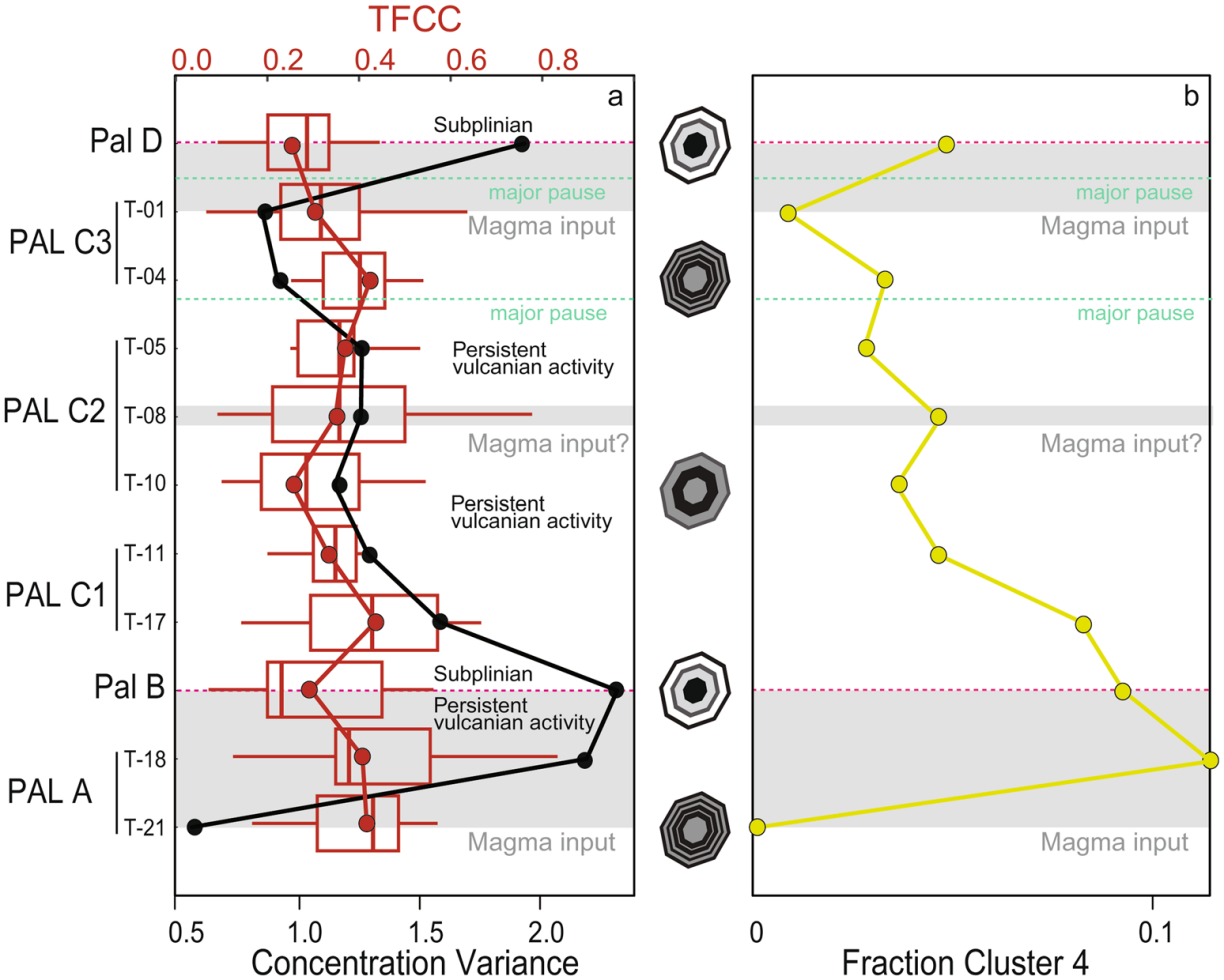
Magmatic inputs along the PEU stratigraphy. (**a**) Chemical variance for each stratigraphic level (σ^2^_tot_, black line) and total fraction of cluster changes (TFCC, red line), see text for explanation; box plots represent the entire range of TFCC recorded by cpx in each level. The schematic cpx sketches indicate the complexity of zoning profiles in response to changes of σ^2^_tot_ and TFCC. (**b**) Fraction of Cluster 4 along the stratigraphy. Grey panels represent the inferred moments of magma input.

In order to quantitatively explore the links between cpx chemistry and magma dynamics, we couple the HC analysis with the analysis of the total concentration variance of chemical elements (σ^2^_tot_) and the total fraction of cluster changes (TFCC; Fig. 8a) along the stratigraphy of PEU. σ^2^_tot_ and TFCC together provide important proxies to trace magmatic processes at depth and are meant here as parameters to investigate the thermal homogenisation of the magma reservoir and the complexity of cpx chemical zoning, respectively, in response to magma recharge events.

The concentration variance (σ^2^) for a given chemical element, is calculated as ref.^60^:

1 $${\upsigma }^{2} = { }\frac{{\mathop \sum \nolimits_{{{\text{i}} = 1}}^{{\text{N}}} \left( {{\text{C}}_{{\text{i}}} - {\upmu }_{{\text{i}}} } \right)^{2} }}{{\text{N}}}{ }$$

where C_i_ is the concentration of the element i, µ_i_ the mean of the element i and N the number of analyses. We calculate the concentration variance for each major oxide (wt.%) in all cpx in each single level and sum it to obtain one value of concentration variance for each level (σ^2^_tot_) (Fig. 8a). We also quantify the complexity of chemical zoning in cpx using the TFCC. This is the number of times that along single profiles a change of cluster is recorded, calculated for all profiles of a specific level and normalised by the total number of analyses collected for each level (Fig. 8a).

While the average chemistry of cpx along the stratigraphy did not show any significant variation (Fig. 3b), several trends emerge when looking together at σ^2^_tot_, TFCC and the fraction of the most mafic cluster 4 (Fig. 8b). Firstly, our analysis highlights some repetitive patterns along the stratigraphy. The deposits of the two sub-Plinian eruptions in the sequence are those that record the highest values of σ^2^_tot_ and relatively low values of TFCC. During the intermediate period of persistent vulcanian activity, σ^2^_tot_ progressively decreases (Fig. 8a). The two sub-Plinian events also erupt cpx with the high fraction of the mafic cluster 4, which drops between the sub-Plinian eruptions (Fig. 8b). Our data shows that the two sub-Plinian events were preceded by a decrease of the TFCC of the cpx crystals, by an increase of σ^2^_tot_ and high fraction of the mafic cluster 4 (Figs. 8a, b). We suggest that these trends recorded by the chemical and textural complexity of cpx crystals, resulted from the protracted input of mafic magma that leads to a diversification of melt chemistry and a thermal homogenisation within the plumbing system in the build-up phase to these two explosive events. The initial magmatic input thus played a major role in the triggering of Pal B rhyolitic eruption, which represents an abrupt change in the eruptive style of this part of the sequence. Petrologic, geochemical and petrographic features of Pal B rhyolite, such as the occurrence of magmatic enclaves and xenocrysts^38^ (see also cpx-melt equilibrium test in Fig. S5; Methods), strongly support the hypothesis that a mafic input and volatiles supply could have triggered an existing shallower rhyolitic reservoir resulting in an explosive eruption.

During the period of persistent vulcanian activity between the two sub-Plinian eruptions, the decrease of σ^2^_tot_ suggests that after the Pal B eruption the input of magma from depth either stopped or decreased. Hence, persistent volcanic activity between Pal C and Pal D was not associated with significant input of deep magma but rather occurred while the shallow portion of the plumbing system was cooling down. In this respect, second boiling or the input of fluids from depth could have triggered the activity during this period^61^.

The top of the sequence is marked by the other sub-Plinian eruption of Pal D. The majority of cpx of Pal D belongs to cluster 3 that is associated to a more evolved magma (Figs. 2c, 6a, b and 7). This is in agreement with the marked different composition of the whole-rock and matrix glass data, mineral phases and melt inclusions of Pal D from the rest of the PEU^38,40,62^. In this respect, petrological and thermodynamic modelling (e.g. ref.^62^) suggests melt differentiation and interaction with a feldspar-rich crystal mush following protracted recharge events and thermal homogenisation of the reservoir to explain the geochemical features of Pal D products, such as the highest K_2_O and Ba contents of Vulcano Island magmas.

### Coupling stratigraphy to magma dynamics

Magma dynamics recognized along the PEU fit well in the general working model of the plumbing system active at Vulcano Island in the last 1000 years, which is dominated by the activity of La Fossa (central system) and Vulcanello (peripheral system), the two main eruptive centres of La Fossa Caldera^44^ (Fig. 1a). Petrologic and chronostratigraphic works suggest that: i) these centres shared a common deep feeding system; ii) the explosive/effusive behaviour of La Fossa was modulated by the contemporaneous (or not) activity of Vulcanello. When both centres were active simultaneously, La Fossa produced mostly explosive activity, while degassed magma fed the Vulcanello effusive phases^27,49,63^. The entire PEU sequence was emplaced after a conspicuous effusive activity at Vulcanello, which occurred contemporaneously to Pal A^27^. The removal of degassed magma during Vulcanello and Pal A activity from the shallower portions of the plumbing system likely promoted the arrival of deep volatile-rich magma feeding the following activity of the PEU.

In the PEU sequence, magmatic input is thus recorded during Pal A, i.e. in the period leading to Pal B (Figs. 8a, b). The tephra sequence belonging to Pal A is in fact characterized by a continuous series of ash layers (Figs. 1c and S1), suggesting the absence of pauses in the eruptive activity. Following the Pal B sub-Plinian event, a decrease in the frequency of the eruptive activity occurs, as testified by a series of stratigraphic unconformities in the stratigraphic succession (Fig. 1c). A subtle magma recharge event could have been registered by the mid deposit of Pal C2 sub-unit (Figs. 8a, b). The progressive decline of magma input from depth is in accordance with the bedding characteristics from Pal C1 to Pal C2 and Pal C3. Bedding records a more intermittent behaviour of the vulcanian activity with time coupled with an increasing duration of eruption pauses (reworking beds interfingered to the primary ash beds in Pal C3, Figs. 1c and S1). The overall characteristics of deposits thus agree with a decline in volatile supply which is in turn in agreement with a decline of the magma supply. Finally, the Pal D eruption, emplaced at the top of the PEU, is characterized by another change in eruptive style following a lava flow and a quiescence period^27^ characterized by another recharge of the magmatic system.

### Final remarks

The application of HC to cpx chemical profiles allowed to recognize at least two periods of relevant magmatic recharge in the PEU sequence. Magma dynamics was further supported with deposit features observed at the outcrop scale, showing that the response of the eruptive activity to changes in the plumbing system can be successfully explored by linking the eruption stratigraphy to detailed crystal data. The approach presented in this work is appropriate to understand similar volcanic successions worldwide constituted by fine-grained material otherwise difficult to investigate. Mineral phases hold crucial information on the magma dynamics and plumbing systems evolution during eruptions. Indeed, the link between magmatic processes occurring in plumbing systems and volcanic record is essential to forecast the future eruptive behaviour, and to correctly decipher monitoring signals at active volcanoes. Our results pose a quantitative base to assess the potential for long-lasting, low-magnitude volcanic activity to transition to larger explosive events. For instance, a decrease of TFCC associated with an increasing proportion of analysis associated to a mafic cluster signal the growth and thermal homogenisation of the subvolcanic reservoir, which could culminate in a significantly larger explosive event. These considerations stress the importance of our approach in unveiling chemical and textural trends that are otherwise hidden in the complexity of the multidimensional nature of mineral chemistry.

We argue that the approach presented in this study, in concert with other techniques such as near-vent lava flows and time series ash sampling (e.g. refs.^64,65^), can boosts the role of petrological monitoring in the long-term forecasting of the eruptive behaviour of volcanic systems undergoing persistent eruptive episodes.

## Methods

### Sampling and samples preparation

Giving the nature of the majority of the PEU products consisting of easily erodible ashes highly susceptible to reworking processes^26^, we studied and sampled the stratigraphic record of the entire explosive sequence (total thickness up to 4 m), in a 13 × 6 m and 2–7 m deep machine excavated trench located in the SE flank of La Fossa cone (Figs. 1a and S1; Supplementary Information; see also ref.^27^).

Clinopyroxene crystals were picked from eleven selected samples of the sequence of the PEU pyroclastic products (Fig. 1c). The samples have been selected in order to at least cover the base and the top of Pal A, Pal C1, Pal C2 and Pal C3. Samples were selected also from the two main pumiceous-lapilli fallout layers of Pal B and Pal D (one sample each). A total of 130 cpx crystals in the grain-size range of 500–1000 µm devoid of alteration and coated in juvenile glass were hand-picked under a stereomicroscope, embedded in resin epoxy mounts. Crystals were preferentially mounted in order to have the c crystallographic axis parallel to the section of the resin mount and polished in order to expose a section through the core of the crystal.

### Scanning electron microscopy and electron microprobe analysis

Backscattered electron images (BSE) of cpx were obtained with a Quanta 450 Field Emission-Scanning Electron Microscope (FE-SEM) installed at the Center for Instrument Sharing at Università di Pisa (CISUP) using a 20 kV filament voltage, 10 mm working distance and 0.1 nA. Major elements core-to-rim profiles of cpx were collected using the electron probe microanalyzer (EPMA) JEOL JXA-8200 superprobe at the University of Geneva using 15 keV acceleration voltage, 10 nA beam current with a 1 µm beam size and 20 µm steps between two analytical spots in a profile. Chemical profiles were obtained systematically by analysing each crystal along core-to-rim transects, perpendicular to the c crystallographic axis and in sections as close as possible to the centre of the crystal, thus minimizing possible compositional variations related to sector zoning of cpx. A total of 130 profiles was collected for a total of 1986 analytical spots (mean analytical total of 100.05 wt.% ± 0.46, see data in the electronic Supplementary Material). The major element chemical composition of the juvenile glass fragments of the PEU samples (data in electronic Supplementary Material) was obtained using an JEOL JXA-8200 superprobe at the Dipartimento di Scienze della Terra at the Università di Milano (operative conditions were 15 kV accelerating voltage and 5 nA beam current). Glasses were analyzed with a defocused electron beam of 5 µm and counting time of 5 s on background and 15 s on peak. The following standards have been adopted for all EPMA analyses: jadeite (Si and Na), labradorite (Al and Ca), forsterite (Mg), andradite (Fe), rutile (Ti), orthoclase (K), barite (Ba), apatite (P) and spessartine (Mn). Na and K were analysed as first elements to minimize alkali loss.

### Hierarchical cluster analysis

We first excluded cpx analyses (n. = 99) with a 3.96 ≤ sum of cations ≤ 4.04 (O = 6). The HC has been conducted on the remaining 1887 analyses using SiO_2_, TiO_2_, Al_2_O_3_, MnO, MgO, FeO, CaO, Na_2_O and excluding those elements with low abundances and high relative variance in cpx (e.g. K_2_O and Cr_2_O_3_). Clinopyroxene analyses were first log-transformed using the isometric log-ratio transformation (ilr)^66^, making them compatible into a Euclidean space (ilr vectors data are reported in electronic Supplementary Material):

2 $$ilr\left( x \right) = { }\sqrt {\frac{i}{i + 1}{ }} { }ln{ }\left[ {\frac{{g(x_{1} ,...,{ }x_{i} }}{{x_{i} + 1}}} \right],{ }i = 1,2,...,D{-}1{ }$$

where, x is a compositional analysis, i is a specific part, D is the number of parts (elements analyzed), and g(x_i_) is the geometric mean of the parts of x^66^. A log-transformation is necessary when dealing with compositional data that are subject to constant-sum closures (i.e., 100 wt.%) and that do not follow a normal distribution. Thus, the use of ilr-transformed data results in a more robust application of statistical analysis algorithms^67^. To prevent any dominance in the ilr data set of some variables having high log-ratio variances (e.g. low abundances and high relative variances) we normalized the log-transformed data set using the column medians and standard deviations following ref.^25^. The normalized ilr data were then used to perform HC following the procedure described in ref.^17^. HC was performed using the “cluster” library in the freeware software R^68^. To cluster the data, we used the Ward linkage method^69^. For a given dataset representable in a Euclidean space, the measurement of variability is calculated through the Euclidean distance of each data point from all the others^69^. Thus, pairs of clusters are gradually identified considering the smallest value of Euclidean distance and combined together. By default the exact number of clusters able to explain a specific problem is a priori unknown. In our case the exact number of clusters representative of the geochemical variance of cpx profiles can be grouped in as many clusters as the data points or in a single cluster containing all of them. Thus, we defined the appropriate number of clusters able at explaining our data through the visual technique of the Elbow Method, by minimizing the total within-cluster sum of square (WSS). The appropriate number of clusters was determined by looking at the total WSS as a function of the number of clusters and choosing a value so that adding another cluster does not significantly improve the total WSS. This visualization indicated that the best number of clusters was 4 (Fig. S2a).

We identified potential outliers in the four clusters following the approach described in ref.^25^ by calculating the Mahalanobis distance, the distance of a given data point and a distribution^70^. The Mahalanobis distance was calculated for each cluster and those values located at distances larger than 3 standard deviation from its centre were identified as outliers. A principal component analysis (PCA) was performed to reduce the dimensions of the ilr-transformed data. Results show that the clusters identified by HC are well defined by PCA. The cluster and relative outliers are better visualized by plotting the first (PCA1) and second (PCA2) principal components (Fig. S2b; data in electronic Supplementary Material). A total number of 160 points were recognized as outliers (Fig. S2c) and not employed in our study, bringing to 1727 analytical spots the number of clustered data used in this work.

An R script, including all the above illustrated steps to perform HC with the user’s own data, is provided as electronic Supplementary Material.

### Clinopyroxene-melt equilibrium

In order to determine the range of temperature and pressure of cpx crystallization, the cpx-melt equilibrium models of refs.^32,71,72^ were used to define equilibrium cpx-melt pairs. Clinopyroxene analyses from the four compositional clusters have been combined with the mean composition of the mafic-intermediate magmas erupted at Vulcano in the same time span of the PEU (i.e. La Fossa-Vulcanello system^44,49,63^; Table S1). Since one of the main problems occurring in the study of fine ash volcanic layers is the lack of whole-rock data that could be assumed as representative of the erupted magma, the cpx analyses have been combined with the mean of whole-rock compositions of Vulcanello shoshonites (clusters 1, 2, 3, 4) and La Fossa latites (clusters 1, 2, 3, 4) and trachytes (only cluster 3) (Table S1). This choice is dictated by the fact that: (i) petrographic observation suggests that the main mineral assemblage of the ash layers (clinopyroxene + plagioclase + olivine) is consistent with that of latitic and shoshonitic magmas of the La Fossa plumbing system; (ii) the composition of the juvenile glass fragments of the PEU shows good correspondence with the residual melt of shoshonitic to latitic Vulcano magmas^38,41,42,44,46,49^.

To verify the chemical equilibrium between the four cpx clusters and melt compositions, the test for equilibrium based on the Fe–Mg exchange coefficient (Kd_Fe-Mg_^cpx-liq^) with an equilibrium range of 0.28 ± 0.08^71^ (Fig. S4a) and the equation of ref.^72^ to calculate the Mg# of the melt in equilibrium with each cpx (Fig. S4b), have been considered. Additionally, we used the equilibrium test of ref.^32^, based on the difference between measured and predicted diopside + hedenbergite cpx components (∆DiHd), assuming in equilibrium cpx-melt pairs with a ∆DiHd < 0.1^34^ (Fig. S5).

In the equilibrium tests, all clusters have been alternately paired with the whole-rock mean compositions of shoshonites (Vulcanello 1 formation, data from^49,62^) and latites (AD 1888–1890 eruption, Pietre Cotte latitic enclaves, data from^38,44,46,73^) (Table S1). Additionally, cluster 3 has been also paired with the mean whole-rock composition of Pal D trachyte (data from^38,46^) (Table S1). This latter choice is dictated by the fact that cluster 3 predominantly occurs in cpx found in the Pal D trachytic eruption. The test for equilibrium based on the Kd_Fe-Mg_^cpx-liq^ (ref. ^71^) (Fig. S4a) shows that all the clusters are in equilibrium with latitic melts except cluster 4 that is in closer equilibrium with a shoshonitic composition. The cluster 3 is also in large part in equilibrium with a trachytic melt. The Mg# of the melt in equilibrium with cpx obtained with the equation of ref.^72^ (Fig. S4b) highlights that cluster 4 is characterized by a wider range of Mg# compared to the other three clusters, being mostly in equilibrium with the shoshonitic melt. Instead, clusters 1, 2 and 3 show a narrower range of Mg#, being thus in equilibrium with lati-trachytic melts. However, it is worth noticing that mafic intermediate magmas erupted at Vulcano in the last 1000 years show similar and partially overlapping values of Mg# (Fig. S4).

The equilibrium test of ref.^32^ shows that all the clusters are in equilibrium with shoshonitic to latitic (clusters 1, 2, 3 and 4) and trachytic (only cluster 3) compositions showing always a ∆DiHd < 0.1 (Fig. S5). In particular, clusters 1 and 2 are in closer equilibrium with a latitic melt having ∆DiHd = 0.007 ± 0.05 and ∆DiHd = 0.014 ± 0.07 respectively. The same clusters, paired with a shoshonitic melt, result still in equilibrium but show slightly higher ∆DiHd of 0.033 ± 0.07 and 0.023 ± 0.07, respectively. Cluster 4 shows a slightly smaller value (∆DiHd = 0.013 ± 0.010) if paired with a shoshonitic melt with respect to a latitic composition (∆DiHd of 0.015 ± 0.012) (Fig. S5). The cpx-melt equilibrium test for cluster 3 yields decreasing ∆DiHd from shoshonite (∆DiHd of 0.043 ± 0.011) to latite (∆DiHd of 0.009 ± 0.020) and trachyte (∆DiHd of 0.008 ± 0.007), resulting in closer equilibrium with a trachytic composition (Fig. SS5). The analyses of the cpx belonging to the rhyolitic Pal B pumice, if paired with a rhyolitic composition get out of the equilibrium range having a ∆DiHd > 1, thus confirming that cpx occurring in the Pal B pumices are xenocrysts belonging to magmas of intermediate composition (see also ref.^38^).

## Supplementary Information

Supplementary Information.

## Data Availability

The data collected for this study and associated R script are available in the electronic Supplementary Material.
