# Supplementary material for: A data driven approach to mineral chemistry unveils magmatic processes associated with long-lasting, low-intensity volcanic activity
Source: Sci Rep. 2023 Jan 24;13:1314. doi: 10.1038/s41598-023-28370-0 (PMC9873939; doi:10.1038/s41598-023-28370-0)
Supplement: Supplementary file 1 — Supplementary Information. [file 41598_2023_28370_MOESM1_ESM.zip › Supplementary Material/Summary of Supplementary material.pdf]

Supplementary material summary information (files uploaded separately):

A data driven approach to mineral chemistry unveils magmatic processes associated with long-lasting, low-intensity volcanic activity

Simone Costa[1*], Luca Caricchi[2], Marco Pistolesi[1], Anna Gioncada[1], Matteo Masotta[1], Costanza Bonadonna[2] & Mauro Rosi[1]

[1] Dipartimento di Scienze della Terra, Università di Pisa, via S. Maria, 53 56126 Pisa, Italy
[2] Department of Earth Sciences, University of Geneva, rue des Maraîchers 13, 1205 Geneva, Switzerland
*simone.costa@dst.unipi.it

Supplementary information: PDF file containing a supplementary text which describes the Stratigraphy of the Palizzi Eruptive Unit and supplementary Figures S1 to S6;

Supplementary table I: Word table with the Average and standard deviation of whole-rock compositional data used in clinopyroxene-melt equilibrium test and thermo-barometric calculations;

Electronic supplementary data: Excel spreadsheet (5 sheets in a same file) with clinopyroxene and juvenile glass EPMA analyses; analyses of cpx used in hierarchical clustering; ilr vectors and PCA component summary;

Hierarchical Clustering script: A folder with an annotated R script to perform clustering along with a txt file of instruction and an example of csv file with input data.
