# Supplementary material for: A data driven approach to mineral chemistry unveils magmatic processes associated with long-lasting, low-intensity volcanic activity
Source: Sci Rep. 2023 Jan 24;13:1314. doi: 10.1038/s41598-023-28370-0 (PMC9873939; doi:10.1038/s41598-023-28370-0)
Supplement: Supplementary file 1 — Supplementary Information. [file 41598_2023_28370_MOESM1_ESM.zip › Supplementary Material/Supplementary information.pdf]

Supplementary information

A data driven approach to mineral chemistry unveils magmatic processes associated with long-lasting, low-intensity volcanic activity

Simone Costa[1*], Luca Caricchi[2], Marco Pistolesi[1], Anna Gioncada[1], Matteo Masotta[1], Costanza Bonadonna[2] & Mauro Rosi[1]

[1] Dipartimento di Scienze della Terra, Università di Pisa, via S. Maria, 53 56126 Pisa, Italy

[2] Department of Earth Sciences, University of Geneva, rue des Maraîchers 13, 1205 Geneva, Switzerland

*simone.costa@dst.unipi.it

Content:

- **Supplementary Text:** The Palizzi Eruptive Unit: Stratigraphic observations of the explosive sequence
- **Figures S1 to S6**

The Palizzi Eruptive Unit: Stratigraphic observations of the explosive sequence

The products investigated in this work belong to the Palizzi Eruptive Unit (Fig. 1) (PEU)[26]. The sequence is variably exposed in several outcrops on the southern flanks of La Fossa volcano, in the Palizzi valley and in the neighboring ring plain at the base of the volcanic cone (Fig. 1). The products of the PEU are particularly relevant in the framework of the last 1000 years of activity of La Fossa volcano. Indeed, during this period, a large volume of volcanic materials was erupted, resulting in a substantial growth of the volcanic edifice[26]. Following this phase of the volcanic history of La Fossa, there is a sharp decrease in the volume of the erupted material[39,44]. The products emitted during the PEU are the results of: i) long-lasting vulcanian activity, ash emissions and minor pyroclastic density currents; ii) lava flows; iii) two sub-Plinian eruptions[28,39]. With the exception of Pal A, dated back to AD ~900–1020 based on ^{14}C analyses on charcoals, and thus being contemporaneous to the Vulcanello 1 lava platform, dated back to AD 900–1040, the entire PEU was emplaced after the activity of Vulcanello 1, given the presence of the Pal B layer directly on top of its lavas[27,39].

Pal A, the lowermost part of the PEU sequence, is a ~50 cm unit constituted by a series of cross-stratified to parallel-bedded, coarse-ash to fine-ash dark grey layers (Fig. S1a, b, c). Pal A is bounded at the base by a grey/brown hard-ground, corresponding to the S1 unconformity of ref.[26], that separates the products erupted in the last 1000 years at La Fossa from the older units. The sequence is abruptly interrupted by the pumiceous fallout layer of Pal B. In the excavated trench (Methods), Pal B is a ~5-10 cm-thick, coarse pale-grey ash layer containing white vesiculated pumiceous lapilli (Fig. S1b, c). The same level, in other outcrops, consists of a massive, clast-supported deposit made up by high vesiculated, white pumiceous bombs and lapilli. The Pal B layer reaches a maximum thickness up to ~1.5 m at the base of the Lentia Dome Complex, at NW of the Fossa Cone (Fig. S1d). Pal C (total thickness of ~2m) has been divided into 3 sub-units on the basis of deposit features. Pal C1, that lies directly above Pal B, consists of several millimetric to centimetric layers made up by parallel bedded fine dark-grey ash (Fig. S1c). Pal C2 is made up by fine ash layers alternating with thin layers of fine pinkish ash and minor

unconformities (Fig. S1e, f). Pal C3, the uppermost part of Pal C, consists of a thin (~5 cm) layer of grey, stratified tephra beds (Fig. S1e, f) with decametric levels of reworked deposits on top, suggesting the presence of a hiatus before the deposition of Pal D. Pal D represents the upper portion of the explosive PEU sequence and it was emplaced after the Pt. Nere lava flow[27]. Pal D consists of a massive, grain-supported deposit bearing pumiceous bombs and lapilli with a thickness up to 2-3 m in medial outcrops, in the southern flanks of La Fossa (Fig. S1a, e, f).

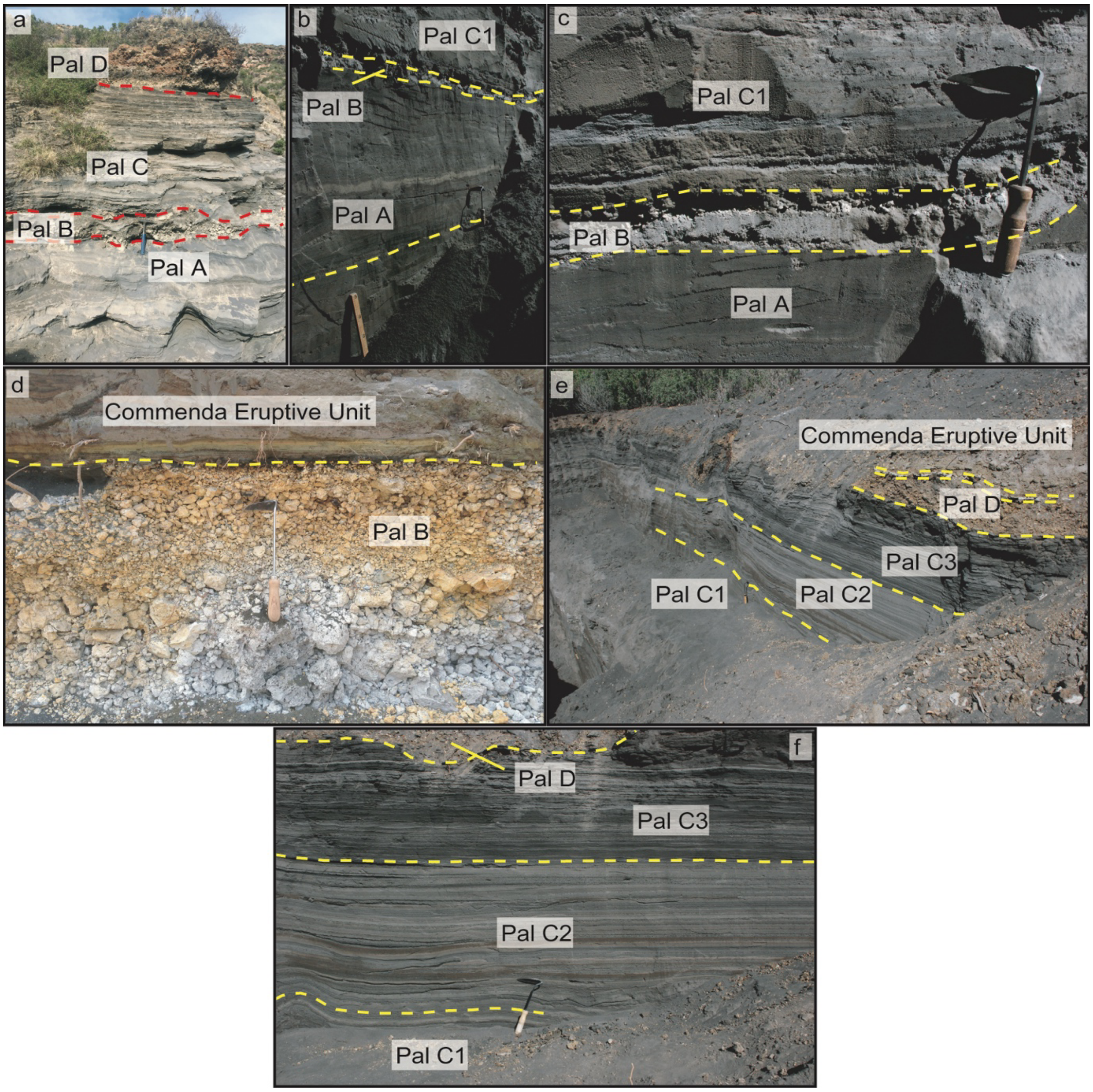

Supplementary Fig. 1 Deposits of the PEU explosive products. a PEU explosive sequence exposed in an outcrop on the southern flank of La Fossa volcano. **b, c, e, f** PEU explosive products in the machine excavated trench. **d** Pal B fallout at the base of Lentia dome complex. Photos a, d were taken by Simone Costa, photos b, c, e, f were taken by Marco Pistolesi.

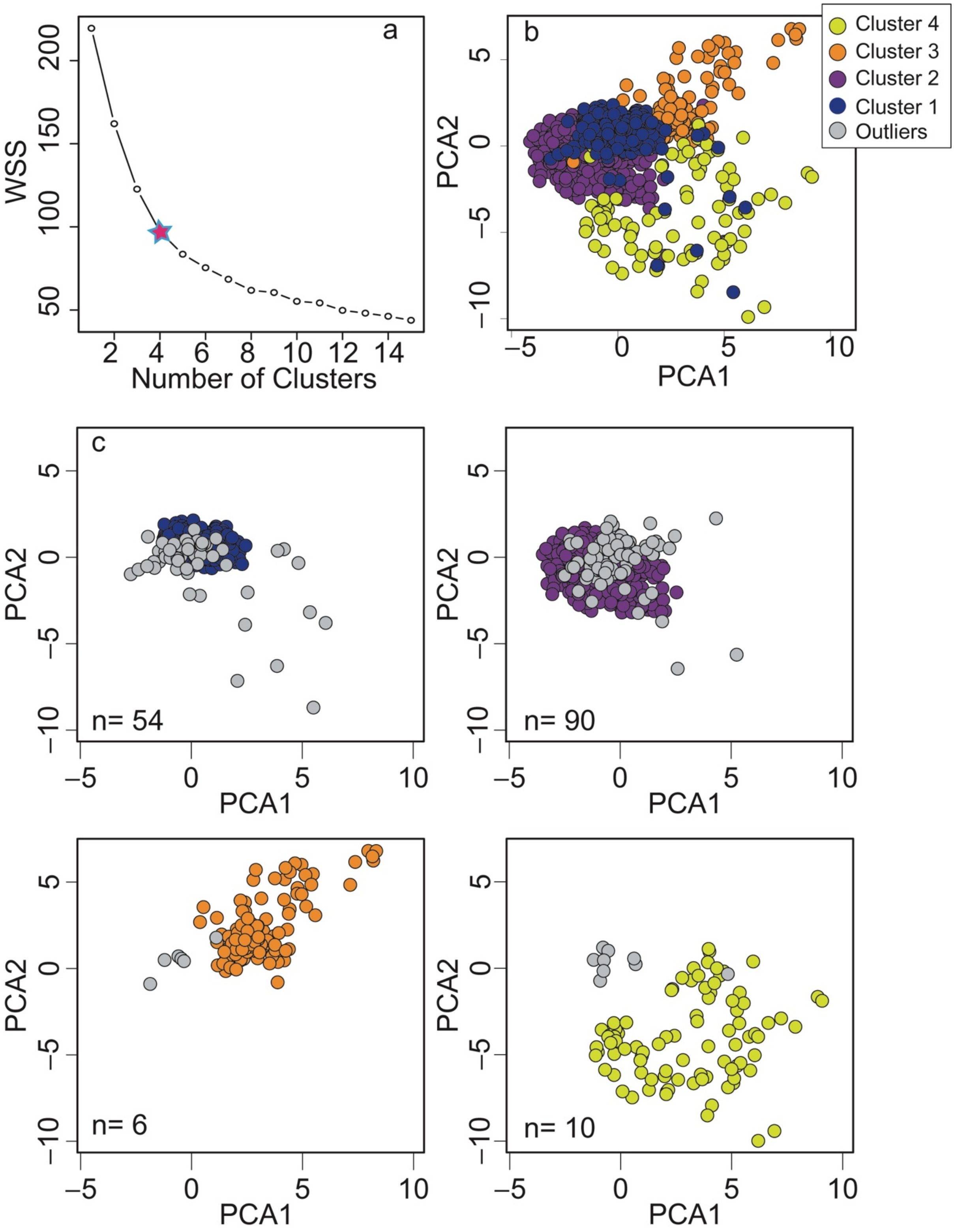

Supplementary Fig. 2. a Visual representation of the Elbow Method in which the appropriate number of clusters is determined by looking at the total within-cluster sum of square (WSS) as a function of the number of clusters and choosing a value so that adding another cluster does not improve the total WSS, accordingly the best number of the cluster was chosen as 4. **b** Clustered data (with outliers included, see Methods) projected using the first (PCA1) and second (PCA2) principal components. **c** PCA1 vs. PCA2 for each cluster with the outliers in grey (Methods), n is the number of outliers for each cluster.

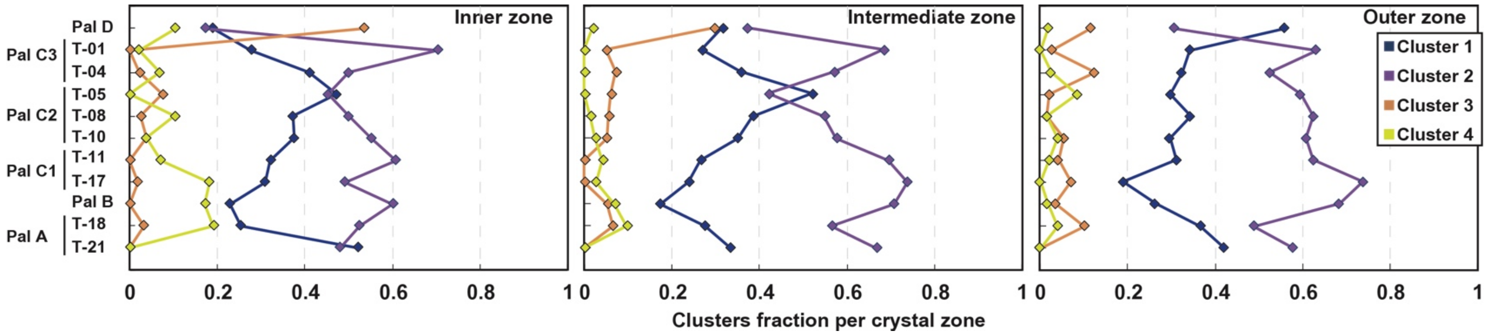

Supplementary Fig. 3. Cluster fractions for the different zones of the cpx crystals.

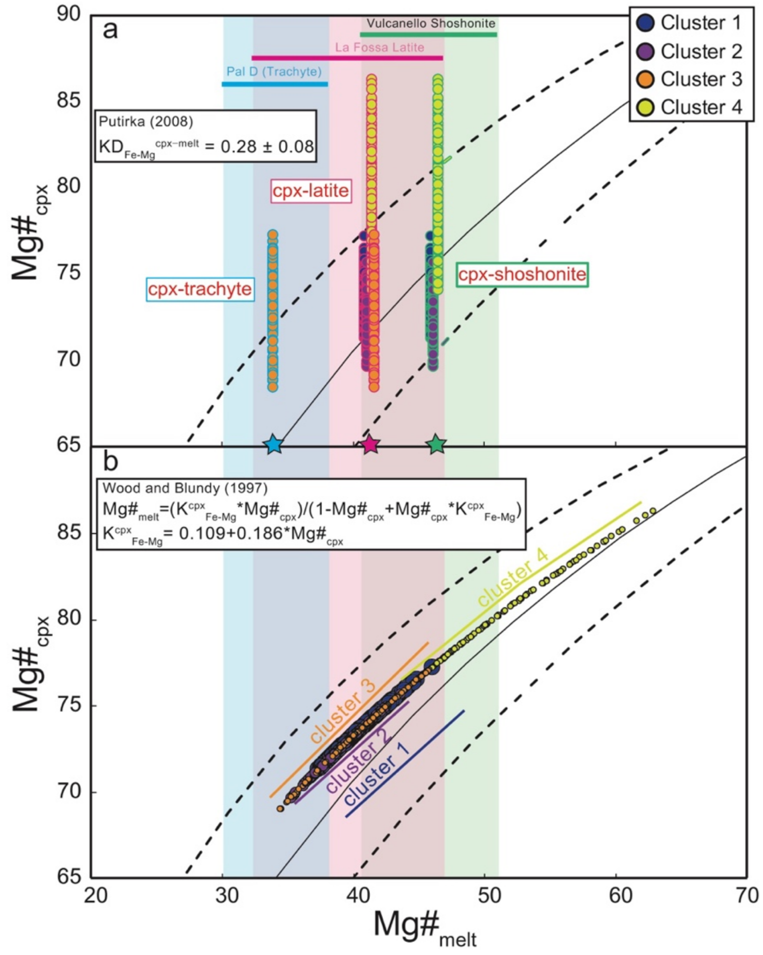

Supplementary Fig.4. Clinopyroxene-melt equilibrium tests. a: equilibrium test based on the Fe-Mg exchange coefficient ($K_D{}^{Fe\text{-}Mg}$cpx-liq)[71] with an equilibrium range of 0.28 ± 0.08; **b:** Mg# of the melt in equilibrium with clinopyroxene clusters obtained with the equation of[72]. Stars indicate the melt composition used for the equilibrium (average of Mg# of shoshonitic, latitic and trachytic products in Supplementary Table 1). The colored bands represent the entire Mg# range of latitic, shoshonitic and trachytic products erupted at La Fossa and Vulcanello in the last 1000 years[38,44,46,49,63,76].

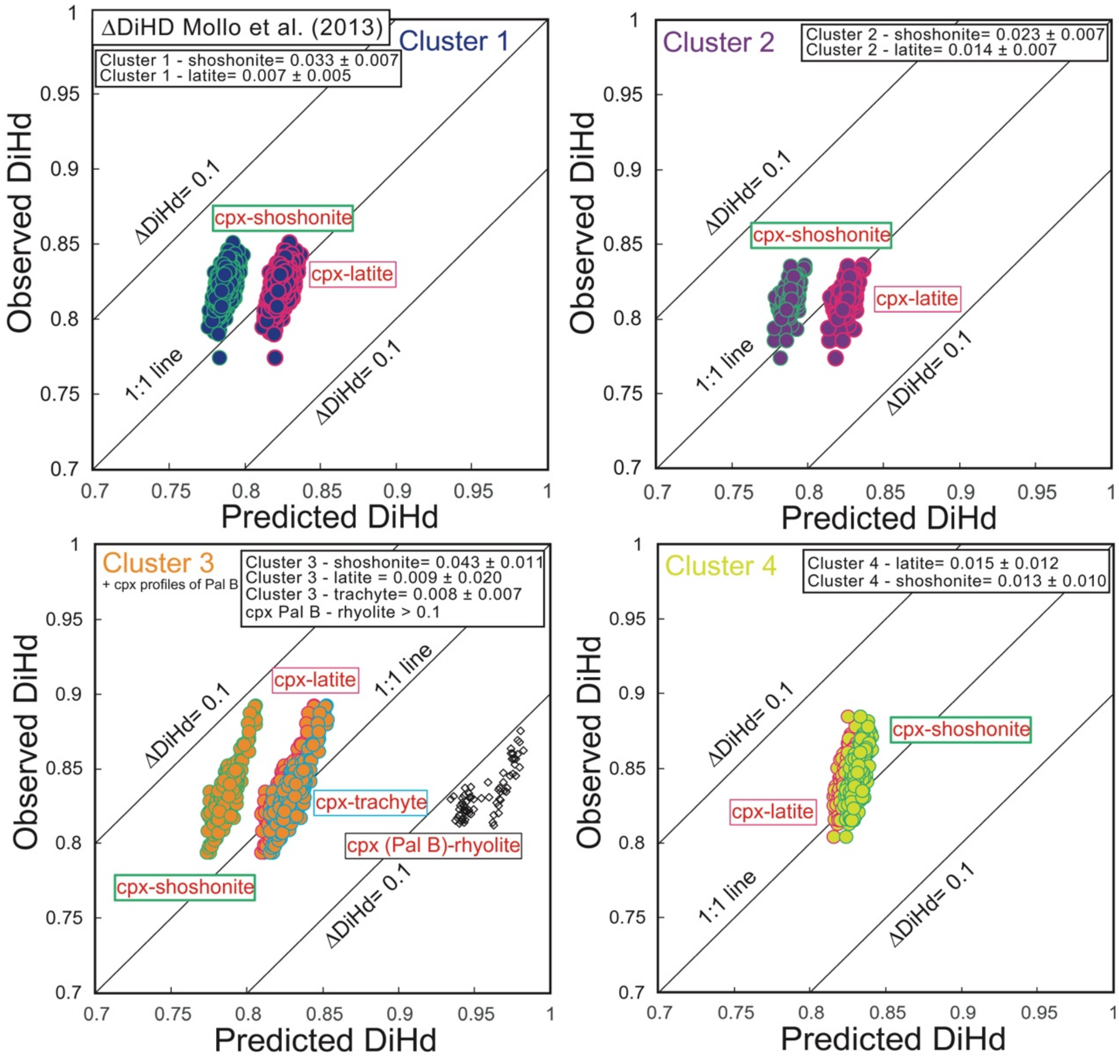

Supplementary Fig. 5. Clinopyroxene-melt equilibrium tests. Test of equilibrium of cpx clusters comparing the observed and predicted values for the DiHd component in clinopyroxene[32]. The melt composition used for the equilibrium, average compositions of whole-rock of shoshonitic, latitic and trachytic products, are in Supplementary Table 1.

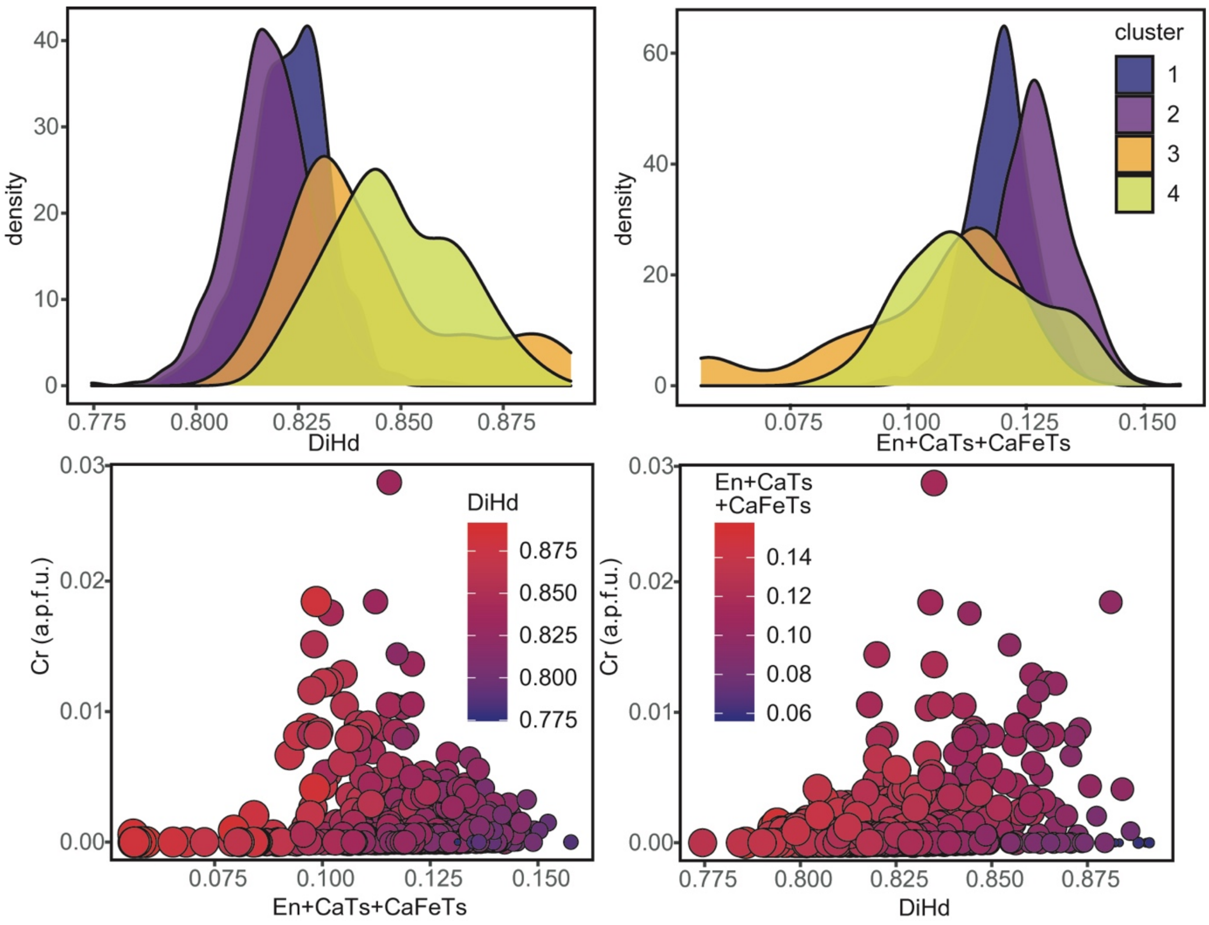

Supplementary Fig. 6. Top panels: Density plots showing the DiHd (left) and the sum of En, CaTs and CaFeTs (Right) for the clinopyroxene clusters. The DiHd decreases while the En+CaTs+CaFeTs increases as a function of increasing undercooling[31]. Down panels: Cr (a.p.f.u., atom per formula units) vs. En+CaTs+CaFeTs and DiHd. Analytical spots showing low undercooling are also enriched in Cr.

References (as cited in the manuscript)

26. Di Traglia, F., Pistolesi, M., Rosi, M., Bonadonna, C., Fusillo, R., Roverato, M. Growth and erosion: The volcanic geology and morphological evolution of La Fossa (Island of Vulcano, Southern Italy) in the last 1000 years. Geomorphology. 194, 94-107 (2013).

27. Malaguti, A. B., Rosi, M., Pistolesi, M., Speranza, F., Menzies, M. The contribution of palaeomagnetism, tephrochronology and radiocarbon dating to refine the last 1100 years of eruptive activity at Vulcano (Italy). Bull. Volcanol. 84, 1-19 (2022).

28. Biass, S., Bonadonna, C., Di Traglia, F., Pistolesi, M., Rosi, M., Lestuzzi, P. Probabilistic evaluation of the physical impact of future tephra fallout events for the Island of Vulcano, Italy. Bull. Volcanol. 78, 37 (2016).

31. Mollo, S., Del Gaudio, P., Ventura, G., Iezzi, G., Scarlato, P. Dependence of clinopyroxene composition on cooling rate in basaltic magmas: implications for thermobarometry. Lithos, 118, 302-312 (2010).

32. Mollo, S., Putirka, K., Misiti, V., Soligo, M., Scarlato, P. A new test for equilibrium based on clinopyroxene–melt pairs: Clues on the solidification temperatures of Etnean alkaline melts at post-eruptive conditions. Chem. Geol. 352, 92-100 (2013).

38. Costa, S., Masotta, M., Gioncada, A., Pistolesi, M., Bosch, D., Scarlato, P. Magma evolution at La Fossa volcano (Vulcano Island, Italy) in the last 1000 years: evidence from eruptive products and temperature gradient experiments. Contrib. Mineral. Petr. 175, 31 (2020).

39. Selva, J., Bonadonna, C., Branca, S., De Astis, G., Gambino, S., Paonita, A., Ricciardi, A. (2020). Multiple hazards and paths to eruptions: A review of the volcanic system of Volcano (Aeolian Islands, Italy). Earth-Sci. Rev. 207, 103186.

44. De Astis, G., Lucchi, F., Dellino, P., La Volpe, L., Tranne, C. A., Frezzotti, M. L., Peccerillo, A. Geology, volcanic history and petrology of Vulcano (central Aeolian archipelago). Geol. Soc. Lond. Mem. 37, 281-349 (2013).

46. Nicotra, E., Giuffrida, M., Viccaro, M., Donato, P., D'Oriano, C., Paonita, A., De Rosa, R. Timescales of pre-eruptive magmatic processes at Vulcano (Aeolian Islands, Italy) during the last 1000 years. Lithos 316, 347-365 (2018).

49. Davì, M., De Rosa, R., Donato, P., Vetere, F., Barca, D., Cavallo, A. Magmatic Evolution and plumbing system of ring-fault volcanism: the Vulcanello Peninsula (Aeolian Islands, Italy). Eur. J. Mineral. 21, 1009-1028 (2009).

63. Fusillo, R., Di Traglia, F., Gioncada, A., Pistolesi, M., Wallace, P. J., Rosi, M. Deciphering post-caldera volcanism: insight into the Vulcanello (Island of Vulcano, Southern Italy) eruptive activity based on geological and petrological constraints. Bull. Volcanol. 77, 76 (2015).

71. Putirka, K. D. Thermometers and barometers for volcanic systems. Rev. Mineral. Geochem. 69, 61-120 (2008).

72. Wood, B. J., Blundy, J. D. A predictive model for rare earth element partitioning between clinopyroxene and anhydrous silicate melt. Contrib. Mineral. Petr. 129, 166-181 (1997).
