# Supplementary material for: A data driven approach to mineral chemistry unveils magmatic processes associated with long-lasting, low-intensity volcanic activity
Source: Sci Rep. 2023 Jan 24;13:1314. doi: 10.1038/s41598-023-28370-0 (PMC9873939; doi:10.1038/s41598-023-28370-0)
Supplement: Supplementary file 1 — Supplementary Information. [file 41598_2023_28370_MOESM1_ESM.zip › Supplementary Material/Supplementary Table 1.docx]

| Unit | Vulcanello 1 |  | Pietre Cotte enclaves and AD 1888-1890 |  | Pal D |  |
| --- | --- | --- | --- | --- | --- | --- |
| Composition | Shoshonite |  | Latite |  | Trachyte |  |
| References | 49, 63 |  | 38, 44, 46, 73 |  | 38, 46 |  |
|  | Average (35) | SD | Average (20) | SD | Average (12) | SD |
| SiO_2_ | 53.95 | 0.52 | 58.80 | 0.83 | 60.21 | 0.90 |
| TiO_2_ | 0.64 | 0.04 | 0.56 | 0.05 | 0.65 | 0.04 |
| Al_2_O_3_ | 16.84 | 0.82 | 16.91 | 1.22 | 17.93 | 0.48 |
| FeO_t_ | 7.85 | 0.45 | 6.23 | 0.51 | 5.10 | 0.77 |
| MnO | 0.15 | 0.01 | 0.13 | 0.01 | 0.11 | 0.01 |
| MgO | 3.67 | 0.56 | 2.46 | 0.38 | 1.45 | 0.24 |
| CaO | 7.27 | 0.78 | 5.20 | 0.58 | 2.97 | 0.43 |
| Na_2_O | 4.60 | 0.74 | 4.02 | 0.16 | 4.31 | 0.28 |
| K_2_O | 4.60 | 0.51 | 5.34 | 0.42 | 6.97 | 0.41 |
| P_2_O_5_ | 0.42 | 0.03 | 0.36 | 0.05 | 0.31 | 0.07 |
|  |  |  |  |  |  |  |

**Supplementary Table 1** Average and standard deviation (SD) of whole-rock compositional data used in clinopyroxene-melt equilibrium test and thermo-barometric calculations (see also Methods section). References as in the manuscript.
