# Supplementary material for: A data driven approach to mineral chemistry unveils magmatic processes associated with long-lasting, low-intensity volcanic activity
Source: Sci Rep. 2023 Jan 24;13:1314. doi: 10.1038/s41598-023-28370-0 (PMC9873939; doi:10.1038/s41598-023-28370-0)
Supplement: Supplementary file 1 — Supplementary Information. [file 41598_2023_28370_MOESM1_ESM.zip › Supplementary Material/Hierarchical Clustering script/README.rtf]

This is the Costa et al script to perform Hierarchical Cluster analysis with mineral data. Paper title: A data driven approach to mineral chemistry unveils magmatic processes associated with long-lasting, low-intensity volcanic activityUsers must install R (https://www.r-project.org/) and R studio (https://www.rstudio.com/products/rstudio/) both of which are free and widely compatible.  As a note: the user’s data file and this script must be kept in the same folder. The work flow goes like this:0- this section must be run first; this install and then call the installed packages1- This section allows to normalize mineral data and to perform isometric log-ratio transformation(the user can modify this section to perform calculation with other mineral data)2- this section is to perform Hierarchical Cluster analysis and Principal Component analysis 3-This section includes a script for  Outliers detectionThe User can then export the results through csv data for plotting contact: simone.costa@dst.unipi.it
